# Venetoclax therapy and emerging resistance mechanisms in acute myeloid leukaemia

**DOI:** 10.1038/s41419-024-06810-7

**Published:** 2024-06-12

**Authors:** Gus O. Nwosu, David M. Ross, Jason A. Powell, Stuart M. Pitson

**Affiliations:** 1 Centre for Cancer Biology, University of South Australia and SA Pathology, Adelaide, SA Australia; 2 Clinical and Health Sciences, University of South Australia, Adelaide, SA Australia; 3 Adelaide Medical School, Faculty of Health Sciences, University of Adelaide, Adelaide, SA Australia; 4 Department of Haematology, Royal Adelaide Hospital, Central Adelaide Local Health Network, Adelaide, SA Australia; 5 Department of Haematology, Flinders University and Medical Centre, Adelaide, SA Australia; 6 School of Biological Sciences, University of Adelaide, Adelaide, SA Australia

**Keywords:** Cancer, Acute myeloid leukaemia

## Abstract

Acute myeloid leukaemia (AML) is a highly aggressive and devastating malignancy of the bone marrow and blood. For decades, intensive chemotherapy has been the frontline treatment for AML but has yielded only poor patient outcomes as exemplified by a 5-year survival rate of < 30%, even in younger adults. As knowledge of the molecular underpinnings of AML has advanced, so too has the development new strategies with potential to improve the treatment of AML patients. To date the most promising of these targeted agents is the BH3-mimetic venetoclax which in combination with standard of care therapies, has manageable non-haematological toxicity and exhibits impressive efficacy. However, approximately 30% of AML patients fail to respond to venetoclax-based regimens and almost all treatment responders eventually relapse. Here, we review the emerging mechanisms of intrinsic and acquired venetoclax resistance in AML and highlight recent efforts to identify novel strategies to overcome resistance to venetoclax.

## Facts

- Venetoclax is a BH3 mimetic small molecule drug that binds and antagonises the pro-survival protein Bcl-2.
- Venetoclax has transformed the front-line treatment of elderly unfit AML patients and is currently undergoing frontline clinical testing in young/fit patients.
- Numerous mechanisms of venetoclax resistance are now emerging including upregulation of the pro-survival Bcl-2 family member Mcl-1.

## Open Questions

- Is overcoming venetoclax resistance achievable to improve long-term clinical responses in AML?
- Can the apparent on-target toxicity of direct Mcl-1 inhibitors be overcome to allow their clinical use to circumvent venetoclax resistance in AML?
- Does indirect targeting of Mcl-1 provide a more tuneable, less toxic avenue for overcoming venetoclax resistance to improve outcomes for AML patients?

## Introduction

Acute myeloid leukaemia (AML) is characterised by the clonal proliferation of immature and functionally impaired haematopoietic cells [1]. The haematopoietic compartment is maintained by a rare population of haematopoietic stem cells (HSCs) [1, 2]. In addition to their self-renewal capacity, HSCs are predominantly quiescent; however, during normal haematopoiesis, they can be activated to produce sub-populations of progenitor cells that generate a hierarchy of committed blood cell lineages [3]. In AML, mutations within HSCs can give rise to pre-leukemic stem cells (pre-LSC) that retain the capacity to undergo normal haematopoiesis, but gain a fitness advantage over normal HSCs resulting in their clonal expansion [4]. Gain of additional mutations by the pre-LSC can result in either impaired haematopoiesis as seen in myelodysplastic syndromes (MDS) [5], overproduction of certain blood lineages as in myeloproliferative neoplasms (MPN) [6], or in the transformation of pre-LSCs into malignant leukemic stem cells (LSC) [7–11]. The founding LSC clone typically acquires additional driver mutations resulting in the outgrowth of a genetically heterogenous sub-clonal population that exhibit a fluid and dynamic architecture during disease progression and treatment [9, 12]. The LSCs can then differentiate to generate a clonally expanded pool of highly proliferative, immature progenitor cells that replace functional blood cells and lead to the symptoms and morbidity associated with AML [13]. Additionally, both the pre-LSC and LSC populations exhibit resistance to conventional chemotherapies and thus can persist to cause disease relapse [11, 14].

## Existing therapies for Aml

### Chemotherapy

Chemotherapy for AML has existed for almost 60 years and remains the frontline treatment for eligible patients. The induction chemotherapy regimen for AML consists of 7 days of a continuous intravenous infusion of the pyrimidine analogue cytarabine alongside 3 days intravenous infusion of the DNA intercalating agents daunorubicin or idarubicin (commonly referred to as “7 + 3” induction) [15]. In AML, the best outcomes for chemotherapy are seen in patients that enter remission and subsequently receive a curative allogeneic HSC transplant [16]. Thus, the ultimate goal of chemotherapy is to eradicate disease and achieve a deep, durable remission [15]. Approximately 70% of AML patients receiving this therapy achieve complete remission; however, the overall survival rate is poor with only 40% of patients surviving at 5-years post treatment [17]. Furthermore, the long-term survival rate for AML patients with adverse-risk cytogenetics is only 20% [17]. Failure to achieve durable remissions may be attributed to the fact that chemotherapy targets proliferating cells and thus often fails to eradicate pre-LSC that can repopulate the LSC population and drive disease relapse [14, 18].

The outlook for elderly or frail patients is very poor, as this population are often ineligible for chemotherapy [19]. For greater than 50% of these patients [20] and for 80–90% patients older than 80 years [21] alternative therapeutic regimens have been historically unsuccessful, until recently. Initially low dose (20 mg/m^2^) cytarabine (LDAC) was trailed as a monotherapy and exhibited limited efficacy, with only 11–19% response rates [22]. To address this unmet clinical need numerous clinical trials have now combined LDAC with additional targeted therapies which has been recently reviewed [23] and discussed below.

### Hypomethylating agents

A characteristic feature of AML is the impaired differentiation of haematopoietic cells. Differentiation arrest arises from excessive genomic hypermethylation that suppresses the expression of genes required for cell maturation [24]. Therefore, drugs that can reverse this hypermethylation have therapeutic potential for AML treatment. Two such drugs are the hypomethylating agents (HMA) decitabine and azacitidine which function by incorporating into DNA and inducing the degradation of DNA methyltransferases (DNMTA), resulting in reduced DNA methylation [25]. Both drugs have been extensively clinically trialled for chemotherapy ineligible AML patients since this cohort have historically lacked effective treatment options [26]. These trials showed that when compared to standard of care (i.e. low/high dose chemotherapy or supportive care) azacitidine modestly improved overall survival (10.4 vs 6.5 months) with an overall response rate (ORR) of 29.9% [27], whereas decitabine did not improve long-term survival but did yield slightly increased ORR (27.7% vs 24%) [28]. Despite only marginally improving treatment outcomes, due to a paucity of superior treatment options, azacitidine and decitabine have each since been adopted as a mainstay treatment for chemotherapy unfit AML patients [26].

### Targeted therapies

The clonal architecture of AML is underpinned by complex interactions between an array of cytogenetic abnormalities and somatic mutations [29]. Advances in sequencing technologies have led to the discovery of a number of molecular abnormalities (Table 1) that contribute to AML pathogenesis. This has facilitated the development and recent therapeutic approval of an array of targeted therapies for the treatment of this disease, including agents targeting Bcl-2, FMS-like tyrosine kinase 3 (FLT3) and isocitrate dehydrogenase (IDH) (Table 2) [30]. Unlike chemotherapies which exert broad genotoxicity, these novel agents selectively target molecular vulnerabilities in AML cells to exploit signalling and epigenetic dependencies required for AML survival [31]. Therefore, targeted therapies offer considerable promise for improving the effectiveness and tolerability of AML therapy, especially in older and unfit adults who may be ineligible for allogeneic stem cell transplant. The emergence of resistance to these agents, however, represents an ongoing clinical challenge. The development, clinical application and resistance mechanisms for FLT3 and IDH inhibitors have been extensively reviewed previously [32, 33]. Here, we describe the development and clinical use in AML of the Bcl-2 antagonist, venetoclax, and focus on the current knowledge of intrinsic and acquired mechanisms of venetoclax resistance in AML, and emerging approaches to combat this.

**Table 1:** Recurring somatic mutations in AML patients.

Mutated gene	Frequency (%)	Cellular/molecular consequence
*NPM1*	27–35	Enhances cytosolic localisation of the nucleophosmin protein [115]
*FLT3*	19–28 (FLT3-ITD) 5–10 (FLT3-TKD)	Constitutive kinase activity either through in-frame duplications of the juxtamembrane domain of the receptor (FLT3-ITD) [116, 117] or point mutations in the tyrosine kinase domain (FLT3-TKD) [118]
*DNMT3A*	26	Inhibits tetramerization and activity of DNA methyl transferase 3 resulting in hypomethylation of regions of the genome [119]
*IDH1/IDH2*	20	Causes isocitrate dehydrogenases to produce the oncometabolite 2-hydroxyglutatrate [120] resulting in impaired TET2 activity and subsequent aberrant genome hypermethylation [121]
*NRAS/KRAS*	13	Constitutive activation of RAS regulated signalling pathways [122]
*RUNX1*	5–10	Impairs DNA binding and transactivation by the RUNX1 transcription factor [123]
*TET2*	8–27	Loss of function mutation resulting in DNA hypermethylation [124]
*TP53*	2–8	Loss of function resulting in impaired tumour suppressive function of TP53 [125]
*CEBPA*	4–6	Expression of a CEBPA isoform lacking a transactivation domain resulting in dysregulated transcriptional activity [126]
*WT1*	6–7	Putative loss of DNA binding [127] and enhanced decay of mutant transcripts [128]
*PTPN11*	4	Putative gain of function due to loss of inter-molecular autoinhibition [129]
*KIT*	2–4	Constitutive activation resulting in aberrant growth signalling [130]
*JAK2*	1–3	Constitutive activation of Jak2 resulting in dysregulated haematopoiesis [131]

**Table 2:** FDA approved targeted therapies for AML.

Therapy	Indication	Approval date	Reference
Midostaurin + chemotherapy	FLT3 + AML	2017	[132]
Gemtuzumab-Ozogamicin + chemotherapy	CD33 + AML CI AML or CD33 + R/R AML	2017	[133]
CPX-351 (Liposomal cytarabine/daunorubicin)	tAML; AML/MDS	2017	[134]
Enasidenib	IDH2 mutant CI AML	2017	[135]
Ivosidenib	IDH1 mutant, CI AML	2018	[136]
Venetoclax + HMA or LDAC	CI AML	2018	[55–57]
Glasdegib + LDAC	CI AML	2018	[137]
Gilteritinib	FLT3 + R/R AML	2018	[138]
CC-486 (oral azacitiditine)	AML in CR or CRi ineligible to undergo intensive curative treatment	2020	[139]

*CI AML* Chemotherapy ineligible AML, *R/R AML* Relapsed/refractory AML, *tAML* therapy related AML, *HMA* Hypomethylating agent, *LDAC* Low dose cytarabine, *CR* Complete remission, *CRi* Complete remission with incomplete hematologic recovery.

## Therapeutic activation of apoptosis

**Fig. 1:**
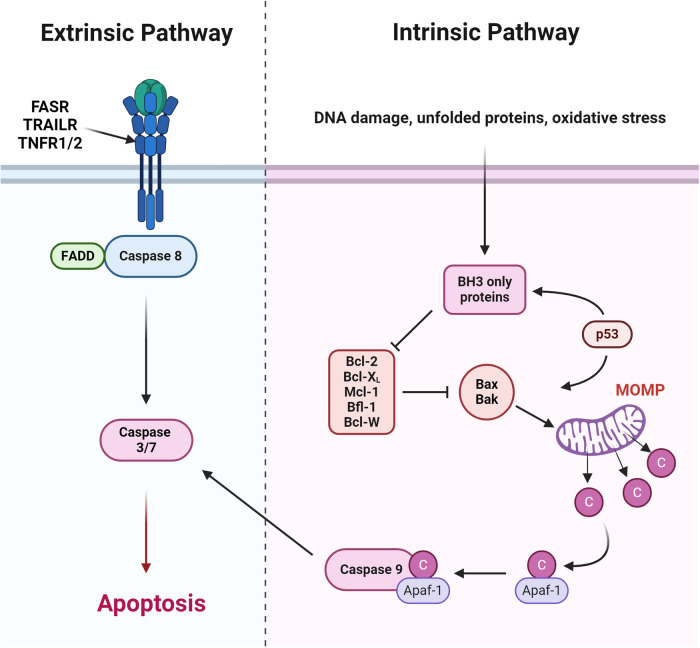
Overview of extrinsic and intrinsic regulation of apoptosis. The extrinsic apoptosis pathway (left) is activated by ligand binding to either the FAS receptor (FASR), TRAIL receptors (TRAILR) or TNF receptor (TNFR) 1 or 2. This results in recruitment of the FAS associated death domain (FADD) that activates caspase 8 which in turn activates caspases 3 and 7 to induce apoptosis. Intrinsic apoptosis (right) is regulated by the Bcl-2 family of proteins. In response to apoptotic stimuli, BH3-only proteins are upregulated and bind to the pro-survival proteins (Bcl-2, Bcl-XL and Mcl-1) to displace Bax and Bak which then cause mitochondrial outer membrane permeabilization (MOMP) resulting in a release of cytochrome C and activation of the apoptosome (a complex containing caspase 9, Apaf-1 and cytochrome c). The apoptosome then catalytically cleaves caspases 3/7 to induce apoptosis. Created with BioRender.

Cancer cells are subject to various chronic cellular stressors including unfolded protein accumulation, heightened metabolic demands, oxidative stress and elevated DNA damage [34]. Healthy cells that fail to resolve such stress normally undergo cell death. In contrast, a common characteristic of cancers is the ability to evade cell death through the dysregulation of programmed cell death mechanisms such as apoptosis [35]. Therefore, lowering the threshold for apoptosis represents a unique and more broadly applicable strategy for the targeted therapy of many cancers, including AML [36]. The two major pathways for apoptosis are the extrinsic and intrinsic pathways (Fig. 1). Through the extrinsic pathway, extracellular signals are detected through either the FAS, TRAIL, TNFα receptors (often called the death receptors), resulting in recruitment of the FAS-associated death domain (FADD) protein and the activation of caspase 8 which subsequently activates caspases 3 and 7 to trigger apoptosis (Fig. 1) [37]. In contrast, the intrinsic pathway of apoptosis is triggered by cell autonomous factors and is regulated by the activity of the Bcl-2 family of proteins which transduce apoptotic stimuli resulting in mitochondrial outer membrane permeability (MOMP), the release of cytochrome c and other pro-apoptotic factors, like DIABLO/SMAC from the mitochondria into the cytoplasm, and subsequent caspase activation and cell death (Fig. 1) [38]. Cross-talk can exist between the extrinsic and intrinsic apoptosis pathways, primarily through death receptor-induced cleavage and activation of the pro-apoptotic protein Bid, which can then promote intrinsic apoptosis [39]. In AML, attenuation of intrinsic apoptosis plays a substantial role in pathogenesis, and so represents an attractive therapeutic target [36].

### Regulation of intrinsic apoptosis

**Fig. 2:**
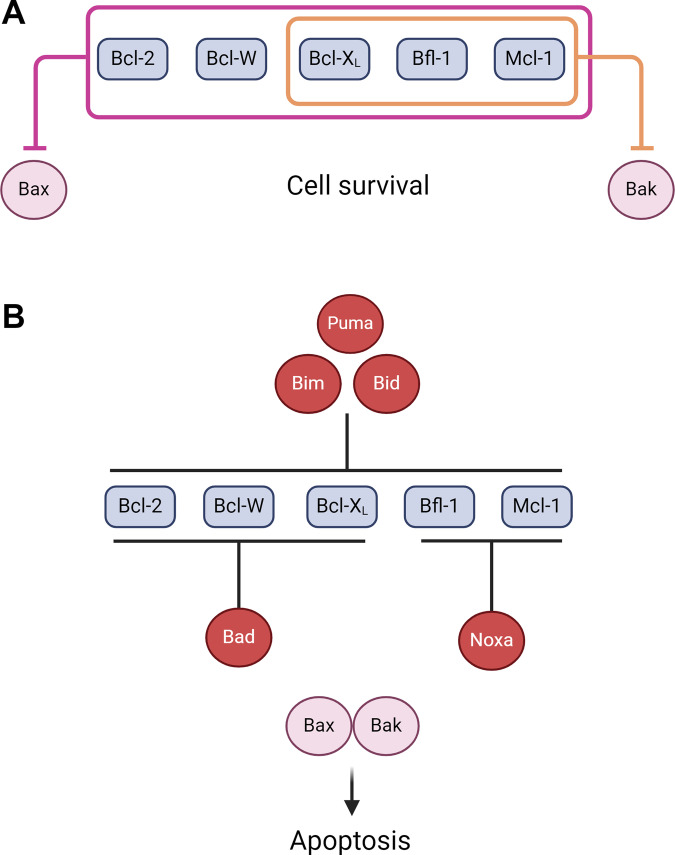
Bcl-2 family regulation of intrinsic apoptosis. **A** In the absence of stress, the prosurvival Bcl-2 proteins bind to and inhibit Bax and Bak to restrain apoptosis. Bcl-2, Bcl-W, Bcl-X_L_, Bfl-1 and Mcl-1 all have inhibitory action against Bax (indicated in purple) whereas only Bcl-X_L_, Bfl-1 and Mcl-1 inhibit Bak (indicated in orange). **B** Stress stimuli upregulates BH3-only proteins such as Puma, Bim, Bid, Noxa or Bad (shown in red) which inhibit the pro-survival proteins by binding to them via their BH3-domains. Puma, Bim and Bid can inhibit all five of these proteins whereas Bad is specific for Bcl-2, Bcl-W and Bcl-X_L_, and Noxa inhibits Bfl-1 and Mcl-1 only. Created with BioRender.

The intrinsic apoptotic pathway is regulated by the Bcl-2 family of proteins which consist of the pro-survival proteins (Bcl-2, Bcl-X_L_, Mcl-1, Bfl-1 and Bcl-W), as well the pro-apoptotic proteins (Noxa, Puma, Bim, Bid, Bad, Bax and Bak) (Fig. 2) [38]. All of the pro-survival proteins and the main effector pro-apoptotic proteins, Bax and Bak, contain four Bcl-2 homology (BH) domains whereas, the other pro-apoptotic proteins possess only the BH3 domain, and are thus known as BH3-only proteins [40]. While minor interpretive variations in the mechanistic details of intrinsic apoptosis exist, the most accepted mechanism involves both the BH3-only proteins and apoptotic effectors interacting with the pro-survival proteins, with the trigger to undergo apoptosis influenced by the relative levels of these proteins [37]. In the absence of apoptotic stimuli, pro-survival proteins are bound with Bax or Bak which prevents them from inducing apoptosis [40]. Upon sufficient stimuli, the activity of pro-apoptotic BH3-only proteins increases, through either post-translational modifications or increases in their transcription, resulting in their binding to the pro-survival proteins via a direct interaction with a hydrophobic region known as the Bcl-2 fold on the pro-survival proteins [41]. Binding of the BH3-only proteins displaces Bax and Bak which then oligomerise to cause MOMP and the subsequent release of cytochrome C, formation of the apoptosome (a complex composed of cytochrome c, caspase 9 and the Apaf-1 protein), activation of caspase 9 and induction of apoptosis (Fig. 1) [38]. Some BH3-only proteins, like Bim, tBid and Puma, can also directly bind and activate Bax and Bak to further enhance MOMP and apoptosis [40]. With the relative abundance or activation of the different Bcl-2 family proteins dictating the trigger for a cell to undergo apoptosis, drugs that inhibit and neutralise pro-survival Bcl-2 proteins or induce the upregulation of the pro-apoptotic proteins have emerged as a rational strategy for targeted therapy.

### BH3-mimetics

The understanding that the BH3-only proteins can bind to pro-survival Bcl-2 family proteins and subsequently stimulate apoptosis has led to the development of a number of small molecule BH3-mimetic drugs (Table 3) to therapeutically induce apoptosis in cancer cells [42]. One of the first to be developed was the compound ABT-737 which binds to Bcl-2, Bcl-X_L_ and Bcl-W to neutralise their anti-apoptotic functions. This compound was shown to induce in vitro killing of follicular lymphoma and chronic lymphocytic leukaemia (CLL) patient samples as well reduce tumour burden in xenograft models of small cell lung carcinoma [43]. However, the therapeutic prospects of ABT-737 were limited since it exhibited poor oral bioavailability, leading to generation of the second-generation compound ABT-263 (navitoclax) [44]. Like ABT-737, navitoclax inhibited Bcl-2, Bcl-X_L_ and Bcl-W and exerted impressive in vitro and in vivo anti-tumour effects but with improved pharmacokinetics which led to follow-up clinical trials of navitoclax for the treatment of CLL [45] and other lymphoid malignancies [46]. However, findings from these trials showed that navitoclax caused dose-limiting on-target toxicity of platelets which ultimately halted its clinical development for lymphoproliferative disorders, [42] although it has shown promising results in the chronic myeloid neoplasm, myelofibrosis [47]. Platelet toxicity has been attributed to the inhibition of Bcl-X_L_ [44, 48] and thus further efforts were made to design a Bcl-2 specific BH3-mimetic which culminated in the development of ABT-199 (Venetoclax) [49]. Venetoclax was shown to potently and selectively target Bcl-2 and induce caspase-dependent apoptosis of acute lymphoblastic leukaemia (ALL) cell lines as well as reduce tumour burden in xenograft models of ALL and B-cell lymphoma [49]. Notably, in vivo experiments demonstrated that venetoclax had minimal effect on platelets in contrast to the on-target toxicity seen with navitoclax [49]. These impressive findings led to a clinical trial of venetoclax for the treatment of relapsed/refractory CLL which showed a 79% overall response rate [45]. Based on this outcome, venetoclax received U.S. FDA approval for the treatment of CLL patients with 17p deletion [42, 50].

**Table 3:** BH3 mimetics.

BH3-mimetic	Target	Target affinity (*K*_*i*_)	Reference
ABT-737	Bcl-2	≤1 nM	[43]
	Bcl-X_L_	≤1 nM	
	Bcl-W	≤1 nM	
ABT-263 (navitoclax)	Bcl-2	<1 nM	[44]
	Bcl-X_L_	≤0.5 nM	
	Bcl-W	0.9 nM	
AZD0466	Bcl-2	N/A	[140]
	Bcl-X_L_	N/A	
APG-1252 (pelcitoclax)	Bcl-2	<1 nM	[141, 142]
	Bcl-X_L_	<1 nM	
ABT-199 (venetoclax)	Bcl-2	<0.01 nM	[45]
S655487/S55746	Bcl-2	N/A	[143]
BGB-11417 (sonrotoclax)	Bcl-2	<1 nM	[144]
APG-2575 (lisaftoclax)	Bcl-2	<1 nM	[145]
FCN-338 (LOXO-338)	Bcl-2	<1 nM	[146]
S63845	Mcl-1	<1.2 nM	[85]
S64315 (MIK665)	Mcl-1	0.026 nM^a^	[87]
AMG-176 (tapotoclax)	Mcl-1	0.06 nM^b^	[89]
AMG-397 (murizatoclax)	Mcl-1	0.015 nM	[90]
AZD5991	Mcl-1	(*K*_*d*_) 0.2 nM^b^	[94]
ABBV-467	Mcl-1	<0.01 nM	[147]
PRT1419	Mcl-1	N/A	[148]

Note: Target affinities measured by fluorescence polarisation except for.

^a^by fluorescence-quenching.

^b^by time resolved fluorescence energy transfer. Target affinities listed as *K*_*i*_ except where noted. N/A, not available.

## Venetoclax in Aml: a backbone for combination therapy

The Bcl-2 protein is known to be overexpressed in AML cells compared to normal hematopoietic cells [51]. This observation coupled with the success of venetoclax-mediated Bcl-2 inhibition in CLL [45] prompted investigation into the possibility of venetoclax as a therapy in AML. Pre-clinical studies revealed that venetoclax exhibited nanomolar potency against both AML cell lines and primary samples and could significantly lower disease burden in cell line and patient-derived xenograft models of AML [52]. These results led to a clinical trial that examined the effect of venetoclax monotherapy in a cohort of relapse/refractory (R/R) AML patients; however, findings from this study showed that venetoclax alone resulted in only modest improvement in patient outcomes with a 19% overall response rate and a short response duration [53]. Follow-up pre-clinical studies investigating dual therapy with venetoclax alongside the HMA azacitidine in ex-vivo AML patient samples revealed that this combination caused synergistic cell death of AML cells [54]. Thus, a phase I clinical trial was launched to assess the effect of combining venetoclax with HMAs (either azacitidine or decitabine) for the treatment of elderly, unfit AML patients [55]. Results from this study showed that venetoclax combined with HMAs resulted in a 73% ORR [55] which was a substantial improvement compared to HMA monotherapy (ORR 28-29%) [27, 28]. The follow up VIALE-A phase III trial confirmed the outcomes for venetoclax plus azacitidine with the combination resulting in an ORR of 66.4% compared to 28.3% in the azacitidine only group [56]. This approach of combining venetoclax with standard of care treatments was then extended to include LDAC elderly chemotherapy ineligible patients associated with poor outcomes [28, 57]. The phase III VIALE-C trial showed that the dual-therapy resulted in improved ORR compared to LDAC alone (48% vs 13% respectively) [57]. Accordingly, this led to the FDA approval of venetoclax in combination with either azacitidine, decitabine or LDAC for chemotherapy-ineligible AML patients. Following this successful outcome, a number of trials have been conducted to assess AML treatment with venetoclax in combination with several other therapies including, gilteritinib (FLT3 inhibitor) in FLT3 mutant R/R AML [58], standard induction chemotherapy in fit AML patients [59] and ivosidenib or ivosidenib plus azacitidine in IDH1 mutant AML [60]. These trials indicated improved outcomes with the dual-therapy approach and support the continued investigation of venetoclax-based regimens for AML. Overall, the approval of venetoclax based regimens (such as venetoclax and azacitidine/decitabine/LDAC) has dramatically improved outcomes for a substantial subset of AML patients. Therefore, the current paradigm sees venetoclax as a backbone for combination therapies appropriate to specific cohorts of AML patients.

## Venetoclax resistance

While AML cells should have heightened sensitivity to venetoclax, given that they upregulate Bcl-2 [51], the minimal effects of venetoclax as a monotherapy for AML are suggestive of intrinsic resistance to the drug [53]. Additionally, approximately 27% of AML patients fail to respond to combined venetoclax and azacitidine therapy and over half of all AML patients that respond eventually relapse [56] which further supports the presence of both intrinsic and acquired resistance to venetoclax. Thus, substantial research has been conducted to determine the molecular mechanisms underpinning venetoclax resistance, with a variety of mechanisms identified.

### Elevated expression of Mcl-1

Venetoclax selectively targets Bcl-2, therefore a shifted dependency on other pro-survival Bcl-2 proteins, like Mcl-1, Bcl-X_L_ or Bfl-1, presents an avenue for mediating resistance to venetoclax [61]. While Bcl-X_L_ plays a prominent role in venetoclax resistance in some lymphoid malignancies, Mcl-1 is the major determinant of venetoclax resistance in AML [62–67]. Early studies showed that pharmacological and genetic blockade of Mcl-1 killed leukemic cells in vitro, significantly reduced in vivo leukemic burden and in a murine model of AML following conditional deletion of Mcl-1, disease relapse only occurred in mice bearing cells that retained Mcl-1 expression [68]. These findings collectively indicate that Mcl-1 functions to promote the sustained survival, growth and pathogenesis of AML cells and highlights a unique dependency for Mcl-1 as opposed to other Bcl-2 proteins in this disease. Further studies also showed that Mcl-1 is the highest expressed Bcl-2 family protein in primary AML samples [69], thus confirming the reliance of AML on this protein.

Pre-clinical studies of venetoclax showed that the OCI-AML3 AML cell line expresses high levels of Mcl-1 (in contrast to other pro-survival Bcl-2 family proteins) and exhibits intrinsic resistance to venetoclax which could be reversed by genetic silencing of Mcl-1 [52], thus demonstrating that Mcl-1 expression can compensate for the inhibition of Bcl-2. Likewise, findings from the initial trial of venetoclax monotherapy in AML indicated that an increased dependency on Mcl-1 in patient samples was a strong predictor of sensitivity and response to venetoclax [53]. AML cells chronically exposed to venetoclax acquire elevated expression of Mcl-1 and increased resistance to venetoclax that similarly could be attenuated by suppression of Mcl-1 [70, 71]. These data collectively indicate that the intrinsic dependency of AML cells on Mcl-1 confers resistance to venetoclax and this resistance can be adaptively increased through further elevation of Mcl-1. Therefore, Mcl-1 is a key target for overcoming venetoclax resistance in AML.

### Genomic instability

Genomic instability is prevalent in CLL patients and often drives mutations in Bcl-2 that render the protein less sensitive to inhibition by venetoclax [72]. These mutations, however, are rarely observed in AML patients, suggesting divergent resistance mechanisms [73].

Acquired mutations in Bax have also been reported in CLL, rendering these tumour cells less sensitive to apoptosis-inducing agents like venetoclax [72]. Notably, recent studies have also reported Bax mutations in 17% of AML patients that relapsed after venetoclax-based therapy [73]. These mutations appear to either result in nonsense-mediated decay of the *BAX* mRNA, truncated or structurally defective Bax protein, or alter the C-terminal α9 helix domain that is critical for Bax localisation to the mitochondrial outer membrane (MOM) necessary for its oligomerisation, MOM pore formation, and initiation of apoptosis [73].

Mutations in the tumour suppressor gene *TP53* are the most frequently observed genetic aberration in cancers [74] and are found in up to 8% of diagnostic AML patients (Table 1) [29]. Furthermore, AML patients possessing *TP53* mutations typically exhibit poor treatment outcomes and resistance to therapy [75]. Indeed, numerous clinical trials of venetoclax alongside HMAs, LDAC or both in AML patients showed that those with mutant *TP53* consistently exhibited inferior ORR (ranging between 0 to 53%) compared to patients with wild-type *TP53* (ORR range of 23 to 71%) [76] which suggests that impaired *TP53* function confers some degree of intrinsic resistance to these treatment regimens. Analyses of venetoclax-HMA or venetoclax-LDAC treated AML patients confirmed the association of *TP53* mutation with refractory disease, and identified the clonal expansion of *TP53* mutant clonal cells in 29-32% of relapsing patients [73, 77]. Further *TP53* knockout studies found that when treated with venetoclax alone or in combination with either decitabine or LDAC, *TP53*^−/−^ AML cells possessed a competitive growth advantage compared to wild-type *TP53* cells [77]. Likewise, AML cells possessing the *TP53* R248W loss of function mutation also exhibited enhanced outgrowth in co-culture with their wild-type counterparts [78]. Similar studies showing that knockout of *TP53* impaired venetoclax-induced apoptotic cell death, indicating a direct role for defective *TP53* in mediating resistance to venetoclax [79]. These findings suggested that lack of *TP53* could prevent venetoclax-induced cell death. However, a closer examination of the role of defective *TP53* in venetoclax resistance revealed that *TP53*^−/−^ AML cells retain sensitivity to venetoclax, but when exposed to sub-optimal concentrations of venetoclax exhibit delayed activation of Bax and Bak, thus increasing their threshold for apoptotic induction [78]. This helps to explain the observed initial activity of venetoclax in the treatment of *TP53* mutant AML with subsequent relapse and outgrowth of *TP53* mutant clones [77] since these cells may have a survival advantage when exposed to insufficient amounts of venetoclax thus allowing them to drive relapse. Accordingly, the mechanism by which *TP53* knockout delays activation of Bax and Bak was attributed to the fact that *BBC3* (Puma), *PMAIP1* (Noxa) and *BIM* are all target genes of *TP53* and are all involved in the activation of Bax/Bak [78].

### Altered metabolic dependency

Contrary to the well described Warburg Effect observed in solid numerous tumours, AML cells are highly reliant on the citric acid cycle and oxidative phosphorylation (OXPHOS) in the mitochondria for energy production. Venetoclax has been demonstrated to decrease OXPHOS by inhibiting the electron transport chain complexes I, II, IV [80]. To overcome this, AML cells generate energy from alternative metabolic pathways including upregulation of the nicotinamide pathway [81] or upregulation of fatty acid oxidation and targeting sensitises cells to venetoclax [82]. In IDH mutant AML the oncometabolite (R)-2-hydroxyglutarate (2-HG) disrupts mitochondrial function by inhibiting cytochrome c oxidase increasing the efficacy on venetoclax [83]. Altered metabolic dependency in response to venetoclax has recently been comprehensively reviewed [72].

## Therapeutically overcoming venetoclax resistance in Aml by targeting Mcl-1

### Direct Mcl-1 inhibitors

The growing recognition of the role for Mcl-1 in mediating venetoclax resistance in AML in addition to the established role of Mcl-1 in the growth of other cancers has led to the development of several Mcl-1 specific BH3-mimetics (Table 3) many of which have entered clinical trials for a range of hematologic malignancies [42, 84].

#### S63845/S64315 (MIK665)

The first Mcl-1 inhibitor with favourable drug properties to be developed was the compound S63845 [85]. This compound was shown to selectively inhibit Mcl-1 and potently kill several AML cell lines in vitro as well as reducing disease burden in a cell line xenograft mouse model [85]. At doses sufficient to kill AML cells, S63845 was shown to have comparably minimal effect on normal hematopoietic cell viability and caused only mild, but tolerable, myelosuppression in mice [85]. However, S63845 displays 6-fold higher affinity for human Mcl-1 compared to mouse Mcl-1 [85] and follow-up studies showed that in mice with humanised Mcl-1, S63845 was poorly tolerated due to significant myelosuppression. These findings cast doubt on the potential therapeutic window for this agent [86] and likely explain why S63845 has not progressed to clinical trials. The developers of S63845 have also created a chemically related Mcl-1 inhibitor, S64315 (MIK665) [87] which has shown in vitro synergy with navitoclax against melanoma cell lines [88]. While there are currently no reports as to the in vivo efficacy of S64315 in humanised Mcl-1 mice, S63415 has progressed to an ongoing phase I/II clinical trial in combination with azacitidine for the treatment of AML (NCT04629443), suggesting this drug may have a more acceptable tolerability profile.

#### AMG-176 (tapotoclax) and AMG-397 (murizatoclax)

Another Mcl-1 inhibitor to be developed is AMG-176 (tapotoclax) which was shown to selectively inhibit Mcl-1 at picomolar concentrations and significantly lowered disease burden in a mouse model of AML [89]. Like S63845, AMG-176 also caused significant myelosuppression in a humanised Mcl-1 mouse model. However, it showed rapid killing of AML cells which allowed for intermittent dosing (1-2 times per week), which if translated to humans, may improve tolerability [89]. AMG-397 (murizatoclax) was derived from AMG-176, and is orally available with enhanced in vitro potency against AML cells and in AML xenografts in mice, when used as a monotherapy or in combination with venetoclax [90]. Both AMG-176 and AMG-397 entered phase I clinical trials for the treatment of multiple myeloma (MM), non-Hodgkin lymphoma, or AML. However, the AMG-397 trial was suspended due to concerns arising from potential cardiac toxicity, leading to suspension of the AMG-197 trial [91]. Therefore, the efficacy and tolerability of AMG-176 and AMG-397 in humans remains unclear. Notably, genetic ablation of Mcl-1 in mice leads to cardiac failure [92, 93], suggesting the observed potential side effects of AMG-397 may be on-target through inhibition of Mcl-1, and a potential issue for all direct Mcl-1 inhibitors.

#### AZD5991

AZD5991 is another novel small molecule Mcl-1 inhibitor, with picomolar binding affinity for Mcl-1 and capacity to induce apoptosis in AML cell lines at sub-micromolar concentrations [94]. AZD5991 caused substantial tumour regression in mouse xenograft studies of human AML [94]. Furthermore, combination of lower doses of AZD5991 with venetoclax greatly suppressed in vivo tumour burden in a subcutaneous xenograft model of human AML in mice [94] suggesting that this combination may have the capacity to overcome resistance to venetoclax. Like S63845, AZD5991 has much (25-fold) greater affinity for human Mcl-1 compared to mouse Mcl-1. Unfortunately, a clinical trial assessing the combination of AZD5991 and venetoclax in R/R AML, MDS, CLL and MM (NCT03218683) was terminated due to potential cardiovascular toxicity [95]. Therefore, the current outlook for AZD5991 as a therapy is unclear.

### Indirect Mcl-1 inhibitors

In addition to the repertoire of direct Mcl-1 inhibitors under development, there are several other compounds that have been shown to downregulate Mcl-1 indirectly through interaction with other cellular targets [91].

#### Deubiquitinase inhibitors

The Mcl-1 protein has a short half-life (approx. 40 min) [96] and Mcl-1 stability is known to be regulated through interaction with deubiquitinases, including Usp9x and Usp24 [97, 98], that protect it from proteasomal degradation. Thus, EOAI3402143, an inhibitor of the deubiquitinases Usp9x and Usp24, reduced Mcl-1 protein levels in MM cells and lowered disease burden in an animal model of MM [98]. Similar pro-apoptotic effects of EOAI3402143 have also been reported with AML cell lines in vitro, both alone and in combination with venetoclax, although the mechanism by which this occurred was ascribed by the authors to destabilisation of mutant FLT3-ITD protein [99].

#### CDK9 inhibitors

Alvocidib (also known as flavopiridol) inhibits multiple CDKs, predominantly functioning through inhibition of CDK9, resulting in global suppression of transcription [100]. Perturbation of transcription is likely to have pronounced effects on cells that are dependent on proteins with short half-lives, such as Mcl-1. Unsurprisingly, alvocidib was shown to reduce Mcl-1 levels in AML cell lines and patient samples and additionally synergised with venetoclax to enhance the killing of AML cells in a xenograft mouse model [101]. Alvocidib progressed to a phase Ib clinical trial in combination with venetoclax for the treatment of R/R AML; however, this drug pairing was found to elicit only marginal improvement in patient outcomes compared to what was previously observed with each agent alone, and has thus not been progressed further [102]. However, given that R/R AML is known to be quite genetically different from de novo AML, this does not preclude testing alvocidib in other AML patient subsets or in different drug combinations. Indeed, alvocidib is currently being trialled in combination with standard induction chemotherapy for the treatment of de novo AML patients and has shown impressive efficacy (69% ORR) [103].

Another promising CDK9 inhibitor is dinaciclib, which has been shown to be more selective and potent than alvocidib with a superior in vivo tolerability [104]. Dinaciclib was shown to kill AML cells possessing MLL::AF9 rearrangements, reduce Mcl-1 transcription and protein levels and extend the survival of mice engrafted with MLL-AF9 leukemia cells [105]. Given the demonstrated ability of dinaciclib to downregulate Mcl-1, the potential for synergy with venetoclax was assessed in a pre-clinical study of diffuse large B cell lymphoma (DLBCL) which showed that dual-therapy with dinaciclib and venetoclax significantly enhanced the survival of mice engrafted with DLBCL cells [106]. Thus, these findings have prompted an ongoing clinical trial assessing dinaciclib in combination with venetoclax for the treatment of R/R AML patients (NCT03484520). It is unclear yet how successful this combination will be; however, the improved selectivity and potency of dinaciclib in contrast to alvocidib could potentially translate to better outcomes with this drug combination.

One of the more recent CDK9 inhibitors to be developed is AZD4573, which was designed to potently and selectively inhibit CDK9 while also having a short in vivo half-life to allow for more flexible and controlled dosing regimens [107]. In contrast to alvocidib and dinaciclib, AZD4573 showed far greater specificity for CDK9 over other CDKs [107]. Additionally, AZD4573 induced rapid Mcl-1 downregulation in the NOMO-1 AML cell line and reduce tumour burden in a NOMO-1 xenograft model [107]. A follow-up study confirmed the selectivity (over 25-fold for CDK9 compared to other CDKs) and potency of AZD4573 and showed that the drug induced rapid Mcl-1 depletion and apoptotic cell death in AML cell lines and dramatically reduced disease burden in an AML cell line xenograft model [108]. The effect of AZD4573 on in vivo disease burden was markedly increased when combined with venetoclax, suggesting synergy between the two agents which provided evidence for combination therapies with these drugs [108]. A clinical trial assessing AZD4573 in combination with venetoclax for the treatment of a variety of R/R hematologic malignancies, including AML, is currently underway (NCT03263637) [91].

PIK-75 was originally described as an inhibitor of phosphoinositide 3-kinase; however, in studies with AML cells, PIK-75 was found to also inhibit the transcriptional kinases CDK7 and CDK9, resulting in reduced *MCL1* transcription and Mcl-1 protein levels [109].

#### Ceramide and activation of the integrated stress response

Sphingolipid metabolism has recently emerged as a key regulator of Mcl-1 [110, 111]. Inhibition of sphingosine kinase 1 (SPHK1) induced cell death in AML cell lines, reduced leukaemic burden in patient-derived xenografts of AML, and sensitised primary AML blasts and leukaemic stem/progenitor cells to chemotherapeutics and venetoclax [110, 111]. Mechanistically, SPHK1 inhibition caused accumulation of ceramide which binds and activates protein kinase R and elicits a non-canonical activation of the integrated stress response (ISR). This ceramide-induced ISR induces transcription of Noxa (*PMAIP1*), a key BH3-only protein that regulates Mcl-1, resulting in Noxa-dependant Mcl-1 degradation. Thus, SPHK1 inhibition, when combined with venetoclax reduces leukemic burden in patient-derived xenografts of AML disease burden in mice.

## Conclusions

Venetoclax-based regimens for AML have been a landmark success in the management of this disease and significant effort is now focussed on developing new therapies that counteract resistance to venetoclax. While several resistance mechanisms exist, the increased dependency on Mcl-1 as a venetoclax resistance factor in AML has highlighted the need for developing drugs to target Mcl-1. Direct Mcl-1 inhibitors have not translated well in human trials, mainly due to their on-target toxicities which appear to severely limit their therapeutic usage. This is consistent with Mcl-1 knockout mice exhibiting lethal cardiac failure [92, 93]. However, mice heterozygous for Mcl-1 display no cardiac pathologies [112] suggesting the potential for inhibitor toxicities to be overcome by reduced dosing.

Indirect Mcl-1 inhibitors are comparatively understudied but, with their apparent better tolerability, may hold the potential to effectively target Mcl-1 while sparing normal tissues and reducing overall toxicities. At present, it remains unclear why indirect Mcl-1 inhibitors appear better tolerated, while still retaining potent activity against AML. It is, however, likely that the impact of these indirect Mcl-1 inhibitors on other pathways plays a role in their anti-AML activity, in synergy with their targeting of Mcl-1. Indeed, inhibition of deubiquitinases, CDK9 or SPHK1 are known to have pro-apoptotic effects that are additional to blocking Mcl-1 [99, 113, 114]. This may allow for induction of AML cell death even with incomplete loss of Mcl-1 function, and thus, avoiding toxicities associated with this. This requires further investigation.

To date, the most advanced indirect Mcl-1 inhibitors have targeted CDK9, but it remains unclear if these drugs will be effective against venetoclax-resistant AML in the clinic. Therefore, to advance the development of indirect Mcl-1 inhibitors, new agents directed against novel cellular targets and pathways that regulate Mcl-1 are urgently needed.
